# Genome Sequence of the Alkaline-Tolerant *Cellulomonas* sp. Strain FA1

**DOI:** 10.1128/genomeA.00646-15

**Published:** 2015-06-18

**Authors:** Michael F. Cohen, Ping Hu, My Vu Nguyen, Nina Kamennaya, Natasha Brown, Tanja Woyke, Nikos Kyrpides, Hoi-Ying Holman, Tamas Torok

**Affiliations:** a Department of Biology, Sonoma State University, Rohnert Park, California, USA; b Earth Sciences Division, Lawrence Berkeley National Laboratory, Berkeley, California, USA; c DOE Joint Genome Institute, Walnut Creek, California, USA

## Abstract

We present the genome of the cellulose-degrading *Cellulomonas* sp. strain FA1 isolated from an actively serpentinizing highly alkaline spring. Knowledge of this genome will enable studies into the molecular basis of plant material degradation in alkaline environments and inform the development of lignocellulose bioprocessing procedures for biofuel production.

## GENOME ANNOUNCEMENT

The cost feasibility of lignocellulosic crops and wastes as feedstocks for biofuel production is limited by the requirement for processing steps that can include an alkaline pretreatment to unbind lignin from the cell wall, followed by acid or enzyme hydrolysis of polysaccharides, and finally fermentation. One possible means to achieve feasibility is through consolidated bioprocessing, whereby chemical processing is facilitated by concomitant metabolic activity of microbial strains capable of functioning throughout the lignocellulose deconstruction process (1). Here, we report the genomic sequence of *Cellulomonas* sp. strain FA1, which possesses desirable characteristics for consolidated bioprocessing, including facultative anaerobic growth, tolerance to high ethanol concentrations and alkaline conditions (pH ≤ 12.2), and catabolism of plant cell wall polysaccharides to ethanol among other products (M. Cohen, M. V. Nguyen, J. Gray, L. Halberg, F. Bernie, A. Hargens, N. Kamennaya, G. Birarda, K. Sasaki, H. Y. Holman, and T. Torok, unpublished results).

Members of the genus *Cellulomonas* are cellulose-degrading Gram-positive rods or coccobacilli (2). There are currently 27 validly named species of *Cellulomonas* (http://www.bacterio.net) and genomes of 8 strains are available in the GenBank database (https://www.ncbi.nlm.nih.gov/genbank/). Strain FA1 was isolated from an alkaline switchgrass enrichment culture inoculated with decaying plant material sampled from within rare ultrabasic calcium hydroxide springs (pH 11.5 to 11.9) at The Cedars, Sonoma County, California (38°37′17.8″N, 123°07′57.0″W), a region of active serpentinization (3).

The genome of strain FA1 was sequenced using the PacBio RS sequencing platform (PacBio, Menlo Park, CA). The generated reads were trimmed and assembled *de novo* using HGAP v2.2.0.p1 (https://github.com/PacificBiosciences/SMRT-Analysis/wiki/SMRT-Pipe-Reference-Guide-v2.2.0). Gene prediction analysis and manual functional annotation was performed within the Integrated Microbial Genomes (IMG) platform (4). The size of the genome of strain FA1 was found to be 3,990,351 bp, comprising 1 scaffold, with a G+C content of 75%. Strain FA1 contained 3,601 predicted genes, 3,540 putative coding sequences (CDS), 6 rRNAs, and 45 tRNAs.

*In silico* DNA-DNA hybridization (DDH) comparisons with GGDC2.0 (http://ggdc.dsmz.de/) (5) between the genome of strain FA1 and other accessible *Cellulomonas* genome sequences in GenBank indicate the closest match to *Cellulomonas flavigena* with an estimated DDH value (high-scoring segment pair length/total length, generalized linear model-based) of 38.5 ± 3.4% while comparisons with other *Cellulomonas* strains gave values from 12.5% to 25.6%. The aligned 16S rRNA gene sequences of these two organisms show 99.3% identity (1,392 bp/1,402 bp).

Strain FA1 has the capacity to metabolize the three major plant cell wall polysaccharides cellulose, hemicellulose, and pectin (M. Cohen, M. V. Nguyen, and T. Torok, unpublished results). Consistent with this, the FA1 genome shows the presence of 82 glycosyl hydrolase and 43 glycosyl transferase homologs, compared to 86 and 44 homologs, respectively, in *C. flavigena*. Four identified Na^+^/H^+^ antiporters encoded in the strain FA1 genome, NhaA, NhaD, NhaP, and the six genes of the multisubunit Mrp system, may have a role in the bacterium’s tolerance to high pH (6, 7).

### Nucleotide sequence accession number. 

This *Cellulomonas* sp. strain FA1 whole-genome shotgun project has been deposited at DDBJ/EMBL/GenBank under the accession no. LBMY00000000.
